# Progress in State Medicine

**Published:** 1902-03-22

**Authors:** 


### Progress in State Medicine.

### SEWAGE AND ITS TREATMENT.

Roechling,[1] reviewing the sewage question during the last century, mentions that Tours in 1825 and Schwann in 1836 were of opinion that organised substances—micro-organisms—played some role in fermentative and putrefactive changes, a theory hotly opposed by Liebig in 1845. Pasteur in 1860, and Koch in 1882, by their individual researches, established the microbic theory, which has been confirmed by the investigations of the Massachusetts State Board of Health, commenced in 1887 and continued to the present time. Although, since 1895 a number of experiments have been made, they have not materially increased our knowledge of the processes taking place in sewage purification. We know that they are of a mechanical, chemical, and biological nature, but as to the processes brought about by these agencies, we know very little beyond the initial and terminal stages. The first Sewage Commission of 1857 decided in favour of land treatment, but the best of land cannot do its work if by systematic neglect and ignorance the essential conditions for successful purification are year by year violated. It was Dibdin who proved that the only natural method of sewage purification was sewage irrigation, and that all other methods were artificial. Public opinion was against sewage farms and nothing but contact bed treatment would do. This led to the Royal Commission on Sewage Purification of 1898. Summing up their conclusions, he says that land treatment has been re-established in its position as the first and only natural method of sewage treatment, and that beside it certain artificial (biological) methods are recognised as, under proper safeguards, admissible for the purification of sewage without land. Referring[2] to the self-purifying powers of soil, he mentions that they must naturally vary considerably with the soil and other local conditions. Soil over-saturated with sewage in time becomes "sewage-sick," and requires a recreative period before it can resume its work of purification. Lime applied to the soil on sewage farms has proved very beneficial in accelerating this process of nitrification. Coarse sewage should be screened or broken up before applying to soil. To ensure a good supply of atmospheric air in the soil on sewage farms systematic under drainage is of primary importance. Discussing the likelihood of pathogenic germs being retained in the soil and ultimately becoming a source of infection to the immediate neighbourhood he states that he has not been able to discover a single instance where a sewage farm has acted as a focus of a local outbreak. On the contrary, during one or two small epidemics of typhoid fever in Berlin, no case of this complaint had been observed on the sewage farm of that city; neither is there any case on record that disastrous results have followed through the passage of pathogenic germs from sewage farms, through the soil into the drains, and thence into streams and rivers. The depth[3] of soil necessary on sewage farms depends on the character and thickness of the top-soil, nature of the cultivation of the top-soil, the character of the sub-soil, and the surface slopes of the land. On some farms a thickness of three feet has proved efficient, and whilst with the exception of peat, no soil is practically wholly inefficient, a loamy, sandy sub-soil has in many instances given every satisfaction. The favourable results obtained with other methods, such as the open or closed septic tanks, have all been achieved from experimental plant only, and these experimental results are hardly likely to be obtained in everyday operations on a large scale. In addition, the plants are now under the care of experts, and can be hardly expected to give such good results when hereafter they are left in the hands of inexperienced men. There can be no doubt that in artificial self-purification carelessness and neglect will work mischief at a much quicker rate than on sewage farms with a fair area. No system will succeed unless scientifically supervised. In the interim[4] report of the Royal Commission on Sewage Disposal, it is stated that it is doubtful whether any land is useless for the purification of sewage, though in the case of stiff clay and peat lands the power to purify sewage seems to depend on the depth of the top soil. Further, the commissioners are satisfied that it is practicable to produce by artificial processes alone, either from sewage or from certain mixtures of sewage and trade refuse, effluents which will not putrefy, which would be classed as good according to ordinary chemical standards, and which might be discharged into a stream without fear of creating a nuisance. In view of this the Local Government Board are recommended, under proper safeguards, to relax the present rule and the application of treated sewage to land be omitted in some cases. They also express their opinion that the general protection of our rivers is a matter of such grave concern that a department should be created which shall be a supreme "rivers authority." Rideal[5] is of opinion that experiments on bacterial purification have now been carried out on a sufficiently large scale to establish the safety of embarking on the treatment of sewage on bacterial lines for even the largest centres of population. Raw sewage, on its arrival at the outfalls, should be roughly screened without previous admixture with any chemicals. Sedimentation may be allowed to take place in open tanks, or channels, in two stages. Sand, road detritus, etc., can thus be first removed, and this may be thrown out upon the land, or dealt with in destructors, without offence; while in the second sedimentation, "sludge," consisting of fœcal and putrescible matter subsides. If these latter substances are simply left in contact with the sewage which constantly flows over them, at least 40 per cent. of the solid sediment disappears by bacterial action. An advantage of this is, that the still impure fluid flowing from the settling tanks has become admirably adapted for undergoing adequate purification in the bacterial coke-beds. The complete cycle of treatment in the London beds consists in filling the coke-bed, emptying it after two hours, leaving its coke contents in contact with the interstitial air for another two hours. It has been found possible to repeat this cycle four times in 24 hours, and, using beds 6 feet in depth, to purify the settled sewage at the rate of 2,000,000 gallons per acre per 24 hours. The final effluent remains entirely inoffensive of smell for an indefinite period in an incubator at summer heat; will maintain the respiration of fish, and would never render water into which it was discharged offensive. Large numbers of bacteria are found in the effluent, but these are useful in effecting, inoffensively, the removal of the organic substances which still remain in the effluent as soon as the effluent mingles with the well-aerated river water. Chemical refuse which is found in the sewage of manufacturing towns seldom exerts any prejudicial effect on the action of the bacteria or upon the coke-beds. The so-called failures to secure bacterial purification of sewage are probably due to the improper construction or working of the bacteria beds. The report[6] of the Manchester Rivers Committee deals with the experimental work carried on in connection with the bacterial treatment of sewage. The present works are designed for the treatment of sewage by chemical precipitation. The sewage as it reaches the works passes through a series of screens and catch-pits to intercept the heavier particles. It is afterwards treated with lime and copperas. These cause the lighter suspended matters to settle in the tanks, the clear effluent flowing into the Ship Canal. The sludge is pushed from the tanks by manual labour into channels leading to two ejectors, from which it is forced under air-pressure into two storage tanks on the banks of the Canal. Thence it flows by gravity into sludge-steamers and is conveyed out to sea. During the five years in which the Roscoe filters have been in active operation the surfaces required attention only occasionally. No material has been renewed. An area of 50 acres of cinder beds is capable of dealing effectively with 30,000,000 gallons of tank effluent per day for five years after the treatment with lime and copperas. The expense is inconsiderable. It may be concluded that the purification on a large scale by chemical precipitation with lime and copperas, and application of the effluent to bacteria beds, is a practical process. The committee report that the method of treatment with septic tanks and bacteria beds will enable them to do away with chemical treatment and lessen the expense of sludge disposal. The final effluent is purer than that obtained from the Roscoe beds. It is calculated that by septic agencies 50 to 60 per cent. of the sludge contained in sewage is dealt with. In regard to the bacteria beds, by working them at a high speed—*i.e.*, filling them frequently during the day without long periods of rest—the effluent may remain good, but the bacterial growth increases so rapidly that the bed becomes spongy and will not allow the water to drain away. They state that it may be anticipated that Manchester sewage, after passing through a system of septic tanks, may be rendered non-putrefactive by treatment on bacteria beds at a rate, allowing for all rests, of 500,000 gallons per acre per day. In the expert's report of 1899 and 1900 it was shown that the filtrate from a secondary bed after septic treatment, contained enough surplus oxygen to oxidise and render inoffensive its own volume of putrid Ship Canal water. Further experiments have since shown that with only one contact the septic tank effluent can be so purified as to be not only itself non-putrefactive, but also capable of purifying a considerable volume of Ship Canal water. The report emphasises the necessity for care in the construction and management of sewage purification works. Creer[7] reports the results of experiments, carried out for the York Corporation, to obtain reliable data as to the various methods of sewage purification. They consisted in the treatment of crude sewage by bacteria beds and ordinary filters, separately and in combination. The first experiment consisted of a closed septic tank, followed by coke or clinker filters. The effluent showed an average of oxygen absorbed, 94 gr. per gallon—just under the Rivers Board provisional standard of purification, and albuminoid ammonia 50 per cent. over the standard. The results were disappointing, this system evidently not being suitable for York sewage. Raw sewage was run through bacteria beds with double contact, as the second experiment, and both in respect of oxygen absorbed and albuminoid ammonia the results were practically within the Rivers Board standard. As compared with chemical filtration this system proved to be very cheap and gave an effluent 56 per cent. purer by the oxygen-absorbed test. A "ladder filter" of 10 chambers gave very unsatisfactory results. An arrangement of open septic tanks and double contact beds also yielded satisfactory results, but an attempt to deal with septic filter effluent by passing it over land proved a failure owing to the unsuitable nature of the soil.

